# Supplementary material for: Reconciling Mining with the Conservation of Cave Biodiversity: A Quantitative Baseline to Help Establish Conservation Priorities
Source: PLoS One. 2016 Dec 20;11(12):e0168348. doi: 10.1371/journal.pone.0168348 (PMC5173368; doi:10.1371/journal.pone.0168348)
Supplement: S1 Dataset — (ZIP) [file pone.0168348.s002.zip › Taxa/Serra Norte/SN_2013/N4WS/Lista N4WS-78.pdf]

CAVIDADE N4WS-0078

Classe	Ordem	Fam/Outros	Gên/Outros	Espécie	S-U	U-S	Zon.
Arachnida	Acari	Argasidae	*Ornithodoros*	sp.1		X	P
Arachnida	Amblypygi	Phrynidae	*Heterophrynus*	*longicornis*	1	1	P
Arachnida	Araneae	Araneidae	*Alpaida*	*septemmammata*	X		E
Arachnida	Araneae	Barychelidae		jovem	1		E
Arachnida	Araneae	Filistatidae		sp.1	X	X	E/P
Arachnida	Araneae	Ochyroceratidae		jovem	X		E
Arachnida	Araneae	Pholcidae	*Leptopholcus*	sp.1	X	X	P
Arachnida	Araneae	Pholcidae	*Mesabolivar*	*eberhard*	X		E
Arachnida	Araneae	Scytodidae	*Scytodes*	jovem	1	1	E
Arachnida	Araneae	Theridiidae	*Achaearanea*	sp.1	X	X	E
Arachnida	Araneae	Theridiosomatidae	*Plato*	sp.1	X		P
Arachnida	Pseudoscorpiones	Olpiidae		sp.1		X	E
Chilopoda	Scolopendromorpha	Scolopocryptopidae	*Newportia*	sp.1	1		E
Entognatha	Collembola	Entomobryidae		sp.6		X	E
Entognatha	Collembola	Paronellidae		sp.1	X		E
Insecta	Blattodea	Polyphagidae		jovem	6	1	P
Insecta	Diptera	Dolichopodidae		sp.	X		E
Insecta	Diptera	Psychodidae	*Sciopemyia*	*sordellii*	X		E
Insecta	Diptera	Tipulidae	Tipulinae	sp.	X		E/P
Insecta	Heteroptera	Lygaeidae	Rhyparochrominae	sp.1		X	E
Insecta	Heteroptera	Reduviidae	Emesinae	jovem	2		E
Insecta	Heteroptera	Reduviidae	Reduviinae	jovem	3	1	E
Insecta	Homoptera	Cercopidae		jovem		X	P
Insecta	Homoptera	Cixiidae		jovem	X		E
Insecta	Hymenoptera	Formicidae	*Pheidole*	sp.2	X		E
Insecta	Hymenoptera	Formicidae	*Wasmannia*	sp.1	X		P
Insecta	Neuroptera	Myrmeleontidae		jovem	2	28	E
Insecta	Orthoptera	Phalangopsidae	*Phalangopsis*	sp.	30	20	P
Insecta	Psocoptera	Psyllipsocidae		jovem		X	E
Amphibia	Anura	Strabomantidae	*Pristimantis*	cf. *fenestratus*	3	3	E/P
Mammalia	Chiroptera	Emballonuridae	*Peropteryx*	sp.	2		P
Mammalia	Rodentia	Cricetidae	*Rhipidomys*	sp.		1	P
